# Four new species of *Dasymallomyia* Brunetti (Diptera, Limoniidae, Chioneinae) from China

**DOI:** 10.3897/zookeys.1275.172279

**Published:** 2026-03-26

**Authors:** Leyou Zhang, Dawei Hong, Bing Zhang, Ding Yang

**Affiliations:** 1 College of Plant Protection, China Agricultural University, Beijing 100193, China School of Chemistry and Chemical Engineering, Xianyang Normal University Xianyang China; 2 College of Plant Science, Xizang Agricultural and Animal Husbandry University, Xizang 86000, China College of Plant Protection, China Agricultural University Beijing China; 3 School of Chemistry and Chemical Engineering, Xianyang Normal University, Xianyang 712000, China College of Plant Science, Xizang Agricultural and Animal Husbandry University Xizang China

**Keywords:** China, Chioneinae, cranefly, key, taxonomy

## Abstract

Previously, nine species of the genus *Dasymallomyia* Brunetti, 1911 were known worldwide, of which three were known from China: *D. clausa* Alexander, 1940, *D. persignata* Alexander, 1932 and *D. signata* Brunetti, 1911. Here, the following four new species of this genus are described from China: *D. bifurcata***sp. nov**., *D. curvispina***sp. nov**., *D. dentata***sp. nov**. and *D. immaculata***sp. nov**. A key to the world species of *Dasymallomyia* is presented.

## Introduction

*Dasymallomyia* Brunetti, 1911 is a small genus of the subfamily Chioneinae with only nine known species, of which four taxa are from the Palaearctic region and six taxa from the Oriental Realm (with one species shared between the regions) (Banerjee et al. 2018; Kato 2022; Oosterbroek 2025). It is characterized by the following features: the general coloration is yellow or fulvous, with polished black pattern on prescutum and presutural scutum; the legs are conspicuously pubescent throughout with long setae; the tibial spur is absent; the wing has an R_3+4_ forked, with R_2_ situated slightly proximal to the fork of R_3+4_, and cell m_1_ is absent; the male outer gonostylus is pale, flat, and forms a long simple lobe while the inner gonostylus is blackened and varies in shape, and the interbase is absent (Brunetti 1911; Alexander 1964; Kato 2022). Furthermore, there is a difference in the wing venation of this genus: the cell 1^st^ M_2_ is usually open, but is closed in *D. clausa* Alexander, 1940 and *D. klapperichi* Alexander, 1955. According to Kania-Klosok et al. (2025), these two species with a closed 1^st^ M_2_ cell form a distinct clade, suggesting that these two species may represent a separate subgenus. However, further evidence is required to substantiate this hypothesis.

*Dasymallomyia* is closely related to the genus *Ellipteroides* Becker, 1907 (Kania-Klosok et al. 2025) and the two genera are highly similar to each other in wing venation, making them one of the most morphologically similar pairs within the subfamily Chioneinae. These two genera can be distinguished from others by the following characteristics: body generally yellow with dark pattern on the prescutum and presutural scutum; legs with setae longer than the diameter of the femur; and R_2_ placed proximal to the fork of R_3+4_ (Brunetti 1911; Kato 2022).

Up to now, three species have been known to occur in China (Brunetti 1911; Alexander 1932, 1940; Kang et al. 2016; Zhang et al. 2023): *D. clausa* Alexander, 1940, *D. persignata* Alexander, 1932 and *D. signata* Brunetti, 1911. In this paper, four new species are added to the fauna of China. A key to the known species of *Dasymallomyia* is presented.

## Material and methods

The specimens were collected using sweep nets or light traps. They were preserved either dry or in 95% ethanol. Subsequently, the specimens were examined and illustrated using a ZEISS Stemi 2000–C Stereo Microscope. Genitalic preparations were made by macerating the apical portion of the abdomen in cold 10% NaOH for 12–15 h. After examination, the preparation was transferred to fresh glycerine and stored in a microvial pinned below the specimen. Types are deposited in the Entomological Museum of China Agricultural University (CAU), Beijing, China.

The morphological terminology mainly follows Cumming and Wood (2017) and de Jong (2017) for wing venation. The nomenclature for the branches (anterior and posterior) of the outer gonostylus follows Kato’ s (2022) description of the “lobe of the gonostylus”. The following abbreviations are used in figures: **aed**, aedeagus; **cerc**, cercus; **goncx**, gonocoxite; **hyp vlv**, hypogynial valve; **i gonst**, inner gonostylus; **o gonst**, outer gonostylus; **ig abr**, anterior branch of inner gonostylus; **ig pbr**, posterior branch of inner gonostylus; **st**, sternite; **tg**, tergite.

## Taxonomy

### Key to world species of *Dasymallomyia* (modified from Alexander, 1964)

**Table:** 

1	Wing with cell 1^st^ M_2_ closed	**2**
–	Wing with cell M_2_ open by atrophy of basal section of vein M_3_	**3**
2	Wing with a conspicuous brown pattern at stigma; outer half of vein R_4_ decurved (Alexander 1940: fig. 7)	***Dasymallomyia clausa* Alexander, 1940**
–	Wing unpatterned; vein R_4_ virtually straight	***Dasymallomyia klapperichi* Alexander, 1955**
3	Wing unpatterned, except for a narrow-darkened seam at cord when this is present (Figs 4D, 6D, 8C)	**4**
–	Wing patterned with conspicuous pale brown (Fig. 1C)	**12**
4	Femora without subterminal ring or inconspicuous (Figs 4A, 4B, 8A)	**5**
–	Femora with conspicuous subterminal ring (Figs 1A, 6A)	**6**
5	Aedeagus with elongate terminal point (Fig. 5F)	***Dasymallomyia curvispina* sp. nov**.
–	Aedeagus with short terminal point (Fig. 9F)	***Dasymallomyia immaculata* sp. nov**.
6	Aedeagus with elongate terminal point	**7**
–	Aedeagus with short to very short terminal point (Figs 2F, 7F)	**8**
7	Posterior branch of inner gonostylus stout with a tooth at base, and with a small tubercle between anterior and posterior branches (Alexander, 1964: fig. 17)	***Dasymallomyia mecophallus* Alexander, 1964**
–	Posterior branch of inner gonostylus slender without tooth at base, and with a strong finger-like lobe near posterior branch (Alexander 1964: fig. 18)	***Dasymallomyia tanyphallus* Alexander, 1964**
8	Apex of gonocoxite without tooth, with setae only; posterior branch of inner gonostylus distinct	**9**
–	Apex of gonocoxite conspicuously toothed (Figs 6D, 6E, 7A, 7B); posterior branch of inner gonostylus reduced (Fig. 7C)	**11**
9	Posterior branch of inner gonostylus with two strong spines (Alexander 1964: fig. 14)	***Dasymallomyia signata* Brunetti, 1911**
–	Posterior branch of inner gonostylus without strong spines	**10**
10	Posterior branch of inner gonostylus with two apical points; outer gonostylus with a small tooth at outer margin (Alexander 1964: fig. 16; Kato 2022: fig. 3)	***Dasymallomyia ditenostyla* Alexander, 1964**
–	Posterior branch of outer gonostylus with three apical points; outer gonostylus without small tooth (Kato 2022: fig. 4)	***Dasymallomyia tachii* Kato, 2022**
11	Outer gonostylus broadened apically (Fig. 7D); the largest tooth on gonocoxite distant from the other spines (Figs 6E, 6F, 7A, 7B)	***Dasymallomyia dentata* sp. nov**.
–	Outer gonostylus slender throughout with narrow apex; all teeth on gonocoxite closely aggregated (Alexander 1964: fig. 15)	***Dasymallomyia compacta* Alexander, 1964**
12	Posterior branch of inner gonostylus unilobed, slender, spine-like (Alexander 1932: fig. 45)	***Dasymallomyia persignata* Alexander, 1932**
–	Posterior branch of inner gonostylus bilobed at base, dorsal lobe with two points and ventral lobe with three points (Fig. 2C)	***Dasymallomyia bifurcata* sp. nov**.

#### Dasymallomyia bifurcata sp. nov.

Taxon classificationAnimaliaDipteraLimoniidae

9FCE7DDE-9B03-5696-8D7D-ECBF217589DE

https://zoobank.org/6D920EAF-5FB5-42CC-8755-4866EEBAD75E

Figs 1, 2, 3

##### Type material.

***Holotype***: China – **Yunnan Prov**. • ♂; Zhaotong; 17 Aug. 2024; Bing Zhang (light trap); CAU. **Paratypes**: – **Yunnan Prov**. • 2 ♂♂, 4 ♀♀; same data as holotype; CAU.

##### Diagnosis.

The species can be recognized by polished prescutum and presutural scutum with black median stripe and lateral spot; scutal lobe each with two black spots. Wing yellowish hyaline, with conspicuous brownish pattern. Outer gonostylus blade-shaped. Inner gonostylus distinctly bifid, posterior branch terminating apically in three points; additionally, a narrow basal lobe ends in two points.

##### Description.

**Male**. Body length 5.3–6.0 mm, wing length 6.2–6.5 mm, antenna length 1.5–1.7 mm.

**Figure 1.**
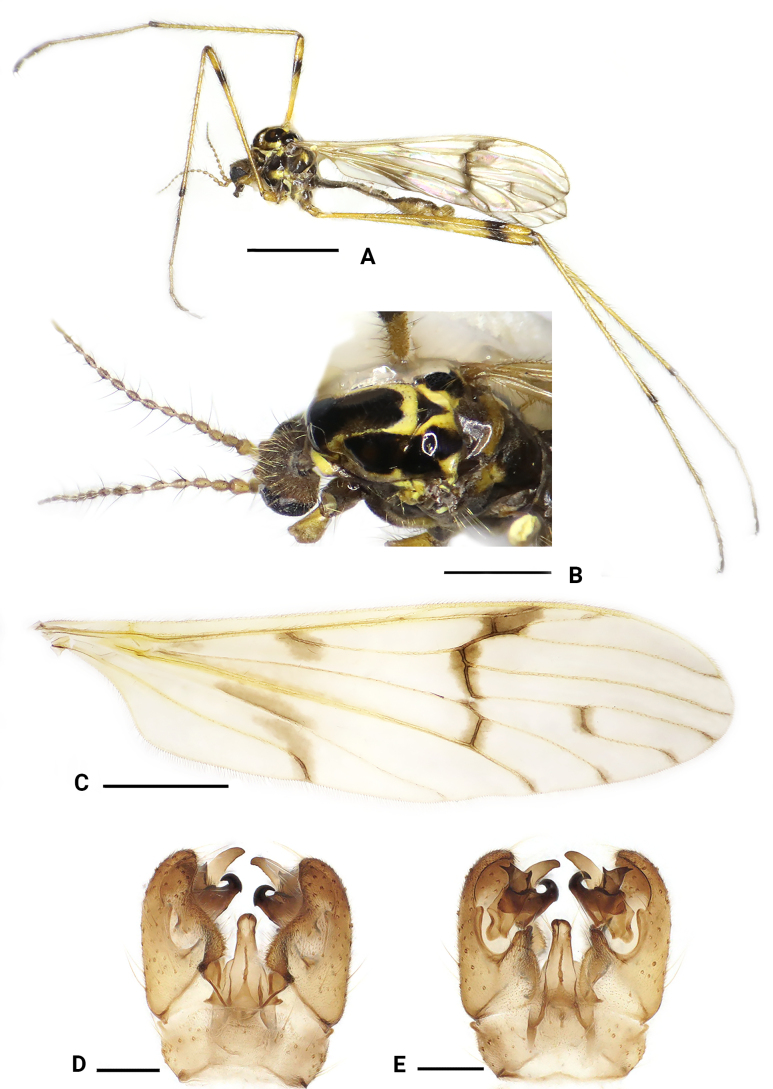
*Dasymallomyia bifurcata* sp. nov. **A**. Male habitus, lateral view; **B**. Head and thorax, dorsolateral view; **C**. Right wing; **D**. Hypopygium, dorsal view; **E**. Hypopygium, ventral view. Scale bars: 2.0 mm (**A, C**); 1.0 mm (**B**); 0.2 mm (**D, E**).

***Head*** (Fig. 1A, B). Mostly brown with pale gray microtrichia. Setae on head yellow to brownish. Antenna brown or weakly bicolored. Scape cylindrical, nearly twice as long as wide; flagellum 14-segmented with long verticils, about 2–3 times as long as segment; flagellomeres oval. Proboscis brownish yellow with brown setae; palpi brownish black with brown setae.

***Thorax*** (Fig. 1A, B). General coloration yellow, distinctly polished on prescutum and presutural scutum. Pronotum brownish yellow. Prescutum and presutural scutum yellow with a black median stripe and one pair of black lateral spots; median stripe distinctly wider on anterior part, posterior margin depressed medially, not reaching transverse suture; lateral spots subtriangular, reaching transverse suture. Postsutural scutum yellow, medially with black triangle; scutal lobe each with two large black spots, partly fused with each other, posterior one distinctly smaller than anterior one. Scutellum dark brown. Mediotergite black, with small yellow area at lateral margin. Pleura with propleuron, anepisternum, anepimeron, ventral half of katepisternum and meron, and laterotergite black to brownish black. Setae on thorax brown. Legs mostly yellow; coxae and trochanters mainly brownish yellow; femora with subterminal brownish ring, distinctly longer than wide; tips of tibiae and tarsi brownish. Setae on legs brownish black. Wing (Fig. 1C) yellowish hyaline, with brownish pattern including a narrow seam extending from stigma along cord; a broader but paler crossband extending from origin of Rs to outer end of vein A, interrupted in cell M; a brownish pattern at fork of M_1+2+3_; small brownish spots at outer end of M_1+2_, M_3_, M_4_ and CuA. Veins yellow, darker in patterned areas. Venation: R_2_ ending at middle of R_3+4_; R_3_ about half as long as R_4_; cell m_1+2_ about as long as its petiole, origin of M_1+2+3_ obtuse and curved, M_3_ distinctly curved at wing margin. Halter 0.7–0.8 mm long, brown except yellow knob.

***Abdomen*** (Fig. 1A). Generally brown, posterior margin of sternites brownish yellow. Setae on abdomen brownish yellow.

**Figure 2.**
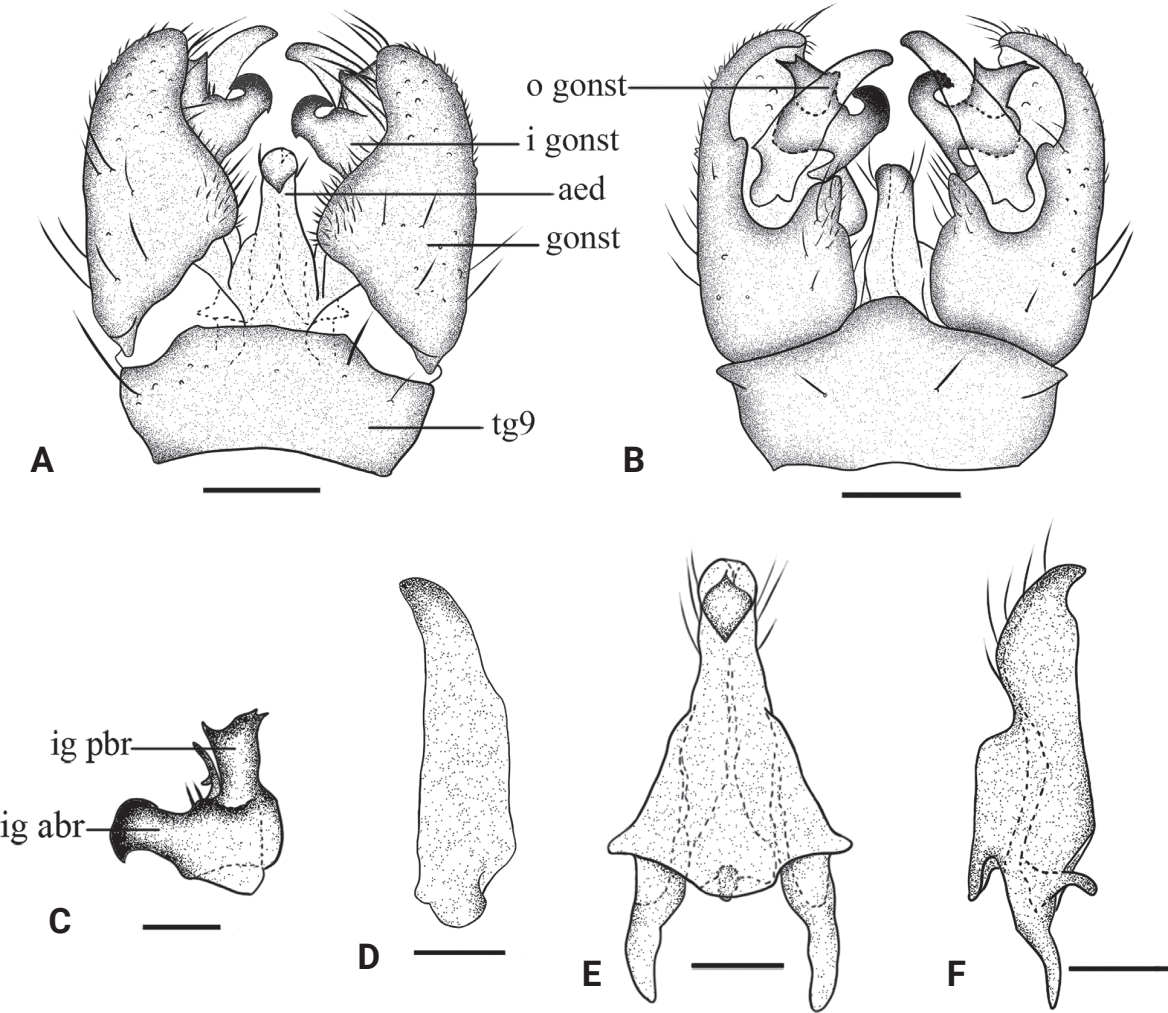
*Dasymallomyia bifurcata* sp. nov. **A**. Hypopygium, dorsal view; **B**. Hypopygium, ventral view; **C**. Inner gonostylus; **D**. Outer gonostylus; **E**. Aedeagal complex, dorsal view; **F**. Aedeagal complex, lateral view. Abbrevations: aed = aedeagus; goncx = gonocoxite; i gonst = inner gonostylus; o gonst = outer gonostylus; ig abr = anterior branch of inner gonostylus; ig pbr = posterior branch of inner gonostylus; tg = tergite. Scale bars: 0.2 mm (**A, B**); 0.1 mm (**C**–**F**).

***Hypopygium*** (Figs 1D, 1E, 2). Posterior margin of tergite 9 slightly convex (Figs 1D, 2A). Sternite 9 strongly convex at middle (Figs 1E, 2B). Gonocoxite distinctly longer than tergite 9, basally broad, rounded at tip (Figs 1D, 1E, 2A, 2B). Outer gonostylus blade-shaped, subhyaline, darkened at tip, slightly reduced at distal third (Fig. 2D). Inner gonostylus distinctly bifid (Fig. 2C); anterior branch apically blackened, sclerotized, curved outward; posterior branch terminating apically into three points, additionally, a narrow basal lobe ends in two points; a small setose tubercle present between anterior and posterior branches. Aedeagus (Fig. 2E, F) nearly conical flask-shaped in dorsal view, short, darkened tip directed posteroventrally, with some setae near tip.

**Female**. Body length 6.2–6.5 mm, wing length 6.5–7.0 mm, antenna length 1.6–1.9 mm. Similar to male in body coloration.

**Figure 3.**
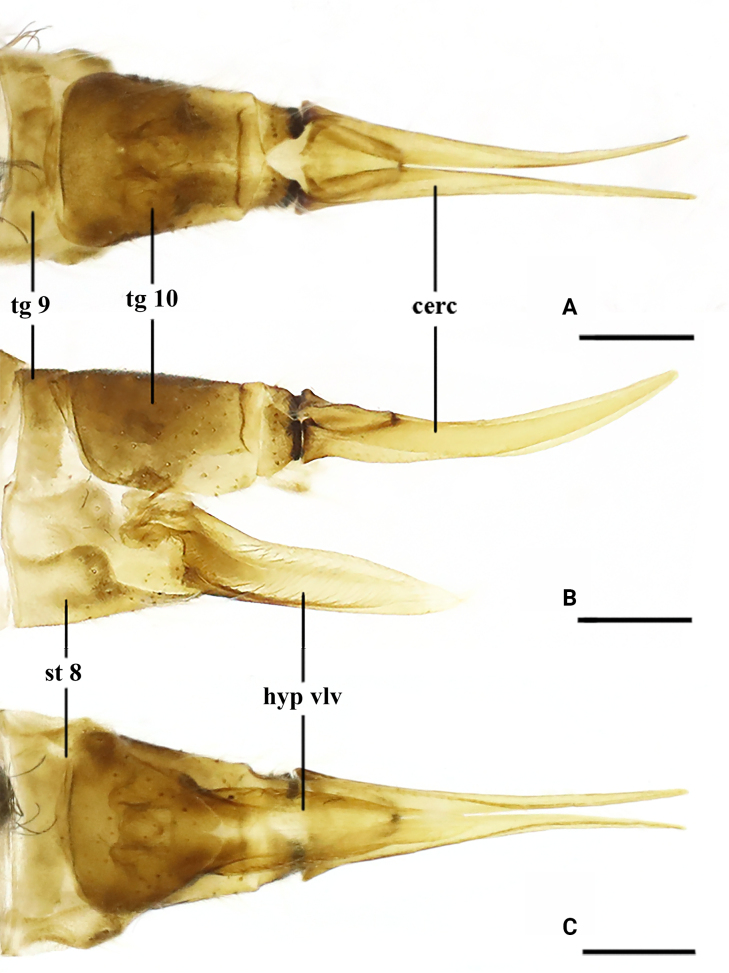
*Dasymallomyia bifurcata* sp. nov. **A**–**C**. Female ovipositor, dorsal, lateral and ventral views. Abbrevations: cerc = cercus; hyp vlv = hypogynial valve; st = sternite; tg = tergite. Scale bars: 0.25 mm.

***Ovipositor*** (Fig. 3A–C). Tergite 9 brown, narrow, slightly convex at caudal margin. Tergite 10 brown, apically blackened, sclerotized, interrupted medially by V-shaped notch. Cercus brownish yellow, tip raised and acute. Hypogynial valve yellow, nearly straight, tip reaching approximately 1/3 of cercus.

##### Distribution.

China, Yunnan.

##### Etymology.

The specific name refers to the bifurcated posterior branch of the inner gonostylus.

##### Remarks.

This species is similar to *D. persignata* in having similar wing venation and some aspects of its patterning, but can be easily separated from the latter by veins M_1+2_, M_3_, M_4_ and CuA with little brownish spots at the wing margin (Fig. 1C). In *D. persignata*, there are no patterns at the above positions (Alexander 1932).

#### Dasymallomyia curvispina sp. nov.

Taxon classificationAnimaliaDipteraLimoniidae

3FB588F0-AC37-5C65-B0D9-F9F8F9007544

https://zoobank.org/BA853282-241A-4C7B-BECE-4DCCDAB5980E

Figs 4, 5

##### Material examined.

***Holotype***: China – **Yunnan Prov**. • ♂; Gongshan, Dulongjiang; 22 May 2007; Xingyue Liu (light trap); CAU.

##### Diagnosis.

Yellowish species. Prescutum and presutural scutum polished, with one brown median stripe and two pairs of brown lateral spots on prescutum, and with two large dark spots on scutal lobe. Wing unpatterned. Abdomen mainly brown. Outer gonostylus blade-shaped, sightly straight. Inner gonostylus dark, distinctly bifid; posterior branch of inner gonostylus long spine-like, with finger-like process near posterior branch.

##### Description.

**Male**. Body length 5.5 mm, wing length 6.2–6.4 mm, antenna length 1.7–1.9 mm.

**Figure 4.**
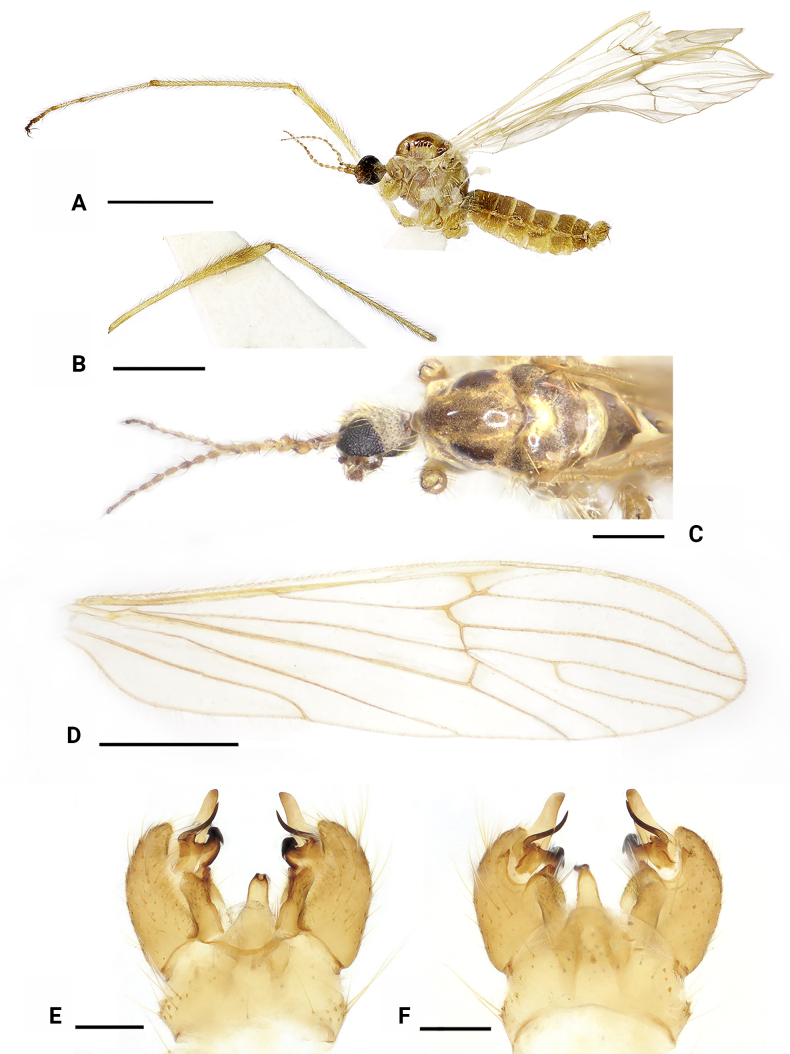
*Dasymallomyia curvispina* sp. nov. **A**. Male habitus, lateral view; **B**. Femur and tibia, lateral view; **C**. Head and thorax, dorsal view; **D**. Right wing; **E**. Hypopygium, dorsal view; **F**. Hypopygium, ventral view. Scale bars: 2.0 mm (**A, B**); 0.5 mm (**C**); 1.0 mm (**D**); 0.2 mm (**E, F**).

***Head*** (Fig. 4A, C). Mostly brownish yellow with pale gray microtrichia. Antenna mostly brownish yellow except scape brown. Scape cylindrical, nearly twice longer than wide; flagellum 14-segmented with long brown verticils, about 2–3 times as long as segment; flagellomeres oval. Proboscis brow with brown setae; palpi brown with brown setae.

***Thorax*** (Fig. 4A, C). General yellow, distinctly polished on prescutum and presutural scutum. Pronotum brownish yellow, with a pale-yellow area posteriorly. Prescutum and presutural scutum yellow with a brown median stripe and two pairs of brown spots near median stripe; median stripe almost of same width in whole length, not reaching transverse suture; anterior lateral spots small, darker, nearly oval, separated from posterior spots. Scutum yellow, with two large subtriangular brown spots on each scutal lobe, narrowly separated from or partly fused with each other, posterior one distinctly smaller than anterior one. Scutellum brown with yellow posterior part. Mediotergite brown. Pleura brown to brownish on propleuron, anepisternum, anepimeron, dorsal half of katepisternum, ventral half of meron and laterotergite. Setae on thorax brownish to yellow. Legs mostly yellow; fore and mid coxae brownish at base; femora with inconspicuous subterminal brownish ring, slightly shorter than wide (Fig. 4B); tips of tibiae and tarsi brownish. Setae on legs brown. Wing (Fig. 3D) yellowish hyaline, unpatterned. Veins mostly brownish, paler on Sc. Venation: R_2_ ending at fork of R_3+4_; R_3_ about 1/3 as long as R_4_, R_4_ decurved at wing margin, cell m_1+2_ about twice as long as its petiole. Halter 0.8–0.9 mm long, pale yellow.

***Abdomen*** (Fig. 4A). Tergites brown with brownish-yellow posterior margin; sternites brownish yellow. Setae on abdomen brownish yellow.

**Figure 5.**
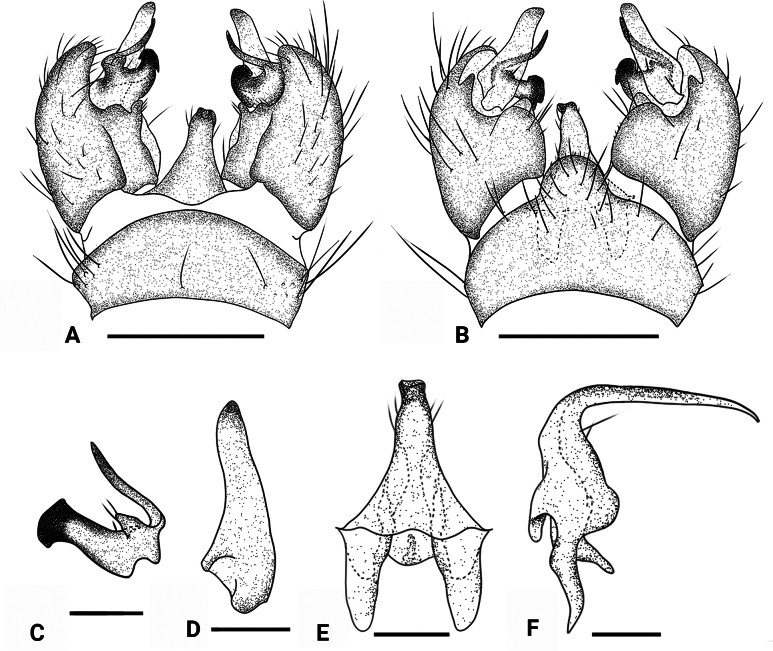
*Dasymallomyia curvispina* sp. nov. **A**. Hypopygium, dorsal view; **B**. Hypopygium, ventral view; **C**. Inner gonostylus; **D**. Outer gonostylus; **E**. Aedeagal complex, dorsal view; **F**. Aedeagal complex, lateral view. Scale bars: 0.2 mm (**A, B**); 0.1 mm (**C**–**F**).

***Hypopygium*** (Figs 4E, 4F, 5). Posterior margin of tergite 9 convex (Figs 4E, 5A. Sternite 9 medially with rounded lobe covered with setae (Figs 4F, 5B). Gonocoxite distinctly longer than tergite 9, broad on basal part, rounded at tip (Figs 4E, 4F, 5A, 5B). Outer gonostylus blade-shaped, sightly straight, darkened at tip (Fig. 5D). Inner gonostylus strongly bifid (Fig. 5C); anterior branch with sclerotized apex curved outward; posterior branch long spine-like, slightly sigmoid, a triangular process present near posterior branch. Aedeagus (Fig. 5E, F) conical in dorsal view, with tip elongated, strongly directed posteroventrally.

**Female**. Unknown.

##### Distribution.

China, Yunnan.

##### Etymology.

The specific name refers to the curved posterior branch of inner gonostylus.

##### Remarks.

This species is similar to *D. tanyphallus* Alexander, 1964 (India, Sikkim) in having an elongate aedeagus. However, it can be distinguished by the following features: in *D. curvispina* sp. nov., the posterior branch of the inner gonostylus is sigmoid, and the elongated tip of the aedeagus is relatively broad; whereas in *D. tanyphallus*, the posterior branch is straight and the elongated tip of the aedeagus is narrow, nearly needle-shaped (Alexander 1964: fig. 18).

#### Dasymallomyia dentata sp. nov.

Taxon classificationAnimaliaDipteraLimoniidae

84DEB420-0F6A-51BD-A4B9-DDFA9D829AEA

https://zoobank.org/88041CEF-9669-4053-8573-F75477308DD9

Figs 6, 7

##### Material examined.

***Holotype***: China – Hubei Prov. • ♂; Shennongjia, Xiaolongtan; 28 Jun. 2023, Jiuzhou Liu (light trap); CAU.

##### Diagnosis.

Yellowish species. Prescutum and presutural scutum polished, with one black median stripe and two pairs of lateral black spots; two large dark spots on each scutal lobe. Wing unpatterned. Abdomen mainly brown. Gonocoxite apically with blackened denticles. Outer gonostylus blade-shaped, shrunken at middle, with darkened, obtuse tip. Inner gonostylus black; anterior branch, short, nearly trapezoidal; posterior branch blackened apically and bent outward, tip with small lateral lobe.

##### Description.

**Male**. Body length 5.5 mm, wing length 6.2–6.5 mm, antenna length 1.7–1.9 mm.

**Figure 6.**
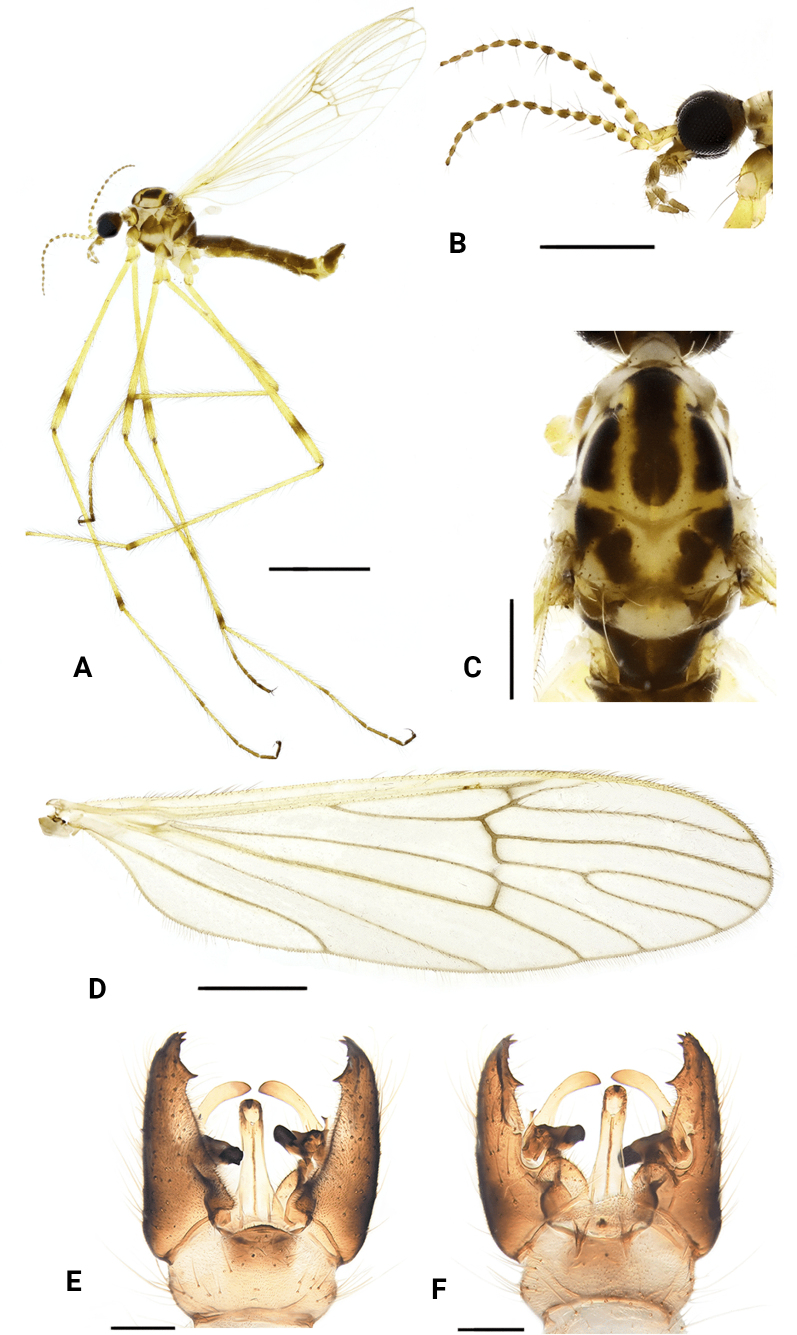
*Dasymallomyia dentata* sp. nov. **A**. Male habitus, lateral view; **B**. Head, lateral view; **C**. Thorax, dorsal view; **D**. Right wing; **E**. Hypopygium, dorsal view; **F**. Hypopygium, ventral view. Scale bars: 2.0 mm (**A**); 1.5 mm (**B**); 0.5 mm (**C**); 1.0 mm (**D**); 0.2 mm (**E, F**).

***Head*** (Fig. 6A, B). Mostly brownish with pale gray microtrichia. Antenna mostly brown except scape and two basal brownish-yellow flagellomeres. Scape cylindrical, nearly twice longer than wide; flagellum 14-segmented with long verticils, about 2–3 times as long as the corresponding segment; flagellomeres oval. Proboscis brownish with brown setae; palpi brownish with brown setae.

***Thorax*** (Fig. 6A, C). Generally yellow, distinctly polished on prescutum and presutural scutum. Pronotum brownish with a pale-yellow area posteriorly. Prescutum and presutural scutum yellow with a brown median stripe and two pairs of brown spots near median stripe; median stripe of almost same width in whole length, not reaching transverse suture; anterior lateral spots small, stripe-shaped, partly fused with posterior spots. Scutum yellow, with two large subtriangular black spots on each scutal lobe, narrowly separated from or partly fused with each other, posterior one distinctly smaller than anterior one. Scutellum brown with a large yellow area in the middle. Mediotergite brown. Pleura brown to brownish on propleuron, anepisternum, anepimeron, dorsal and ventral margins of katepisternum, ventral half of meron and laterotergite. Setae on thorax brown. Legs mostly yellow; fore coxa and trochanter mainly brownish; femora with subterminal brownish ring nearly as long as wide; tips of tibiae and tarsi brownish. Setae on legs brownish black. Wing (Fig. 6D) yellowish hyaline, unpatterned. Veins mostly brownish, paler on Sc and base of vein R. Venation: R_2_ ending at fork of R_3+4_; R_3_ about 1/3 as long as R_4_; cell m_1+2_ about twice as long as its petiole, origin of M_1+2+3_ obtuse and curved. Halter 0.8–0.9 mm long, pale yellow.

***Abdomen*** (Fig. 6A). Generally brown, posterior margin of sternites brownish yellow. Setae on abdomen brownish yellow.

**Figure 7.**
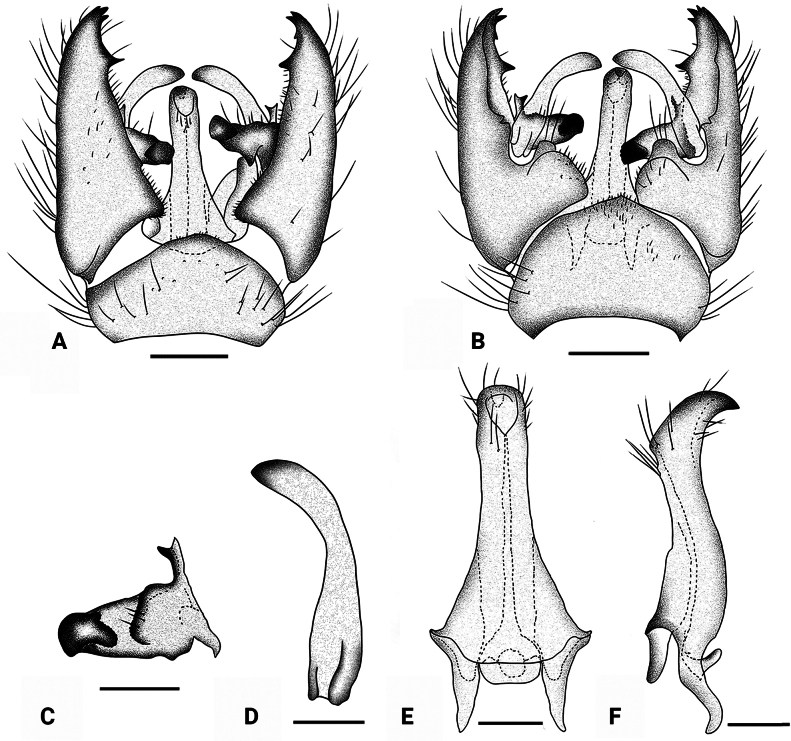
*Dasymallomyia dentata* sp. nov. **A**. Hypopygium, dorsal view; **B**. Hypopygium, ventral view; **C**. Inner gonostylus; **D**. Outer gonostylus; **E**. Aedeagal complex, dorsal view; **F**. Aedeagal complex, lateral view. Scale bars: 0.2 mm (**A, B**); 0.1 mm (**C**–**F**).

***Hypopygium*** (Figs 6E, 6F, 7). Posterior margin of tergite 9 slightly convex (Figs 6E, 7A). Sternite 9 strongly convex at middle (Figs 6F, 7B). Gonocoxite longer than tergite 9, basally broad, tip with one larger preapical and three smaller apical blackened denticles (Figs 6E, 6F, 7A, 7B). Outer gonostylus blade-shaped, subhyaline, darkened at tip, strongly shrunken at middle (Fig. 7D). Inner gonostylus weakly bifid (Fig. 7C); anterior branch sclerotized, distinctly elongated, nearly trapezoidal; posterior branch blackened apically and bent outward, its apex bearing a distinct, small lateral lobe. Aedeagus (Fig. 7E, F) subconical in dorsal view, its short, darkened tip directed posteroventrally, with some setae near tip.

**Female**. Unknown.

##### Distribution.

China (Hubei).

##### Etymology.

The specific name refers to the toothed gonocoxite.

##### Remarks.

This new species is similar to *D. compacta* Alexander, 1964 (India, Sikkim) in the general morphology of the gonocoxite and inner gonostylus. It differs, however, in the following characters: the outer gonostylus broadens apically, whereas in *D. compacta* it remains slender throughout with a narrow apex; on the gonocoxite, the largest spine is set farthest from the other spines, while in *D. compacta* all spines are closely aggregated (Alexander 1964: fig. 15).

#### Dasymallomyia immaculata sp. nov.

Taxon classificationAnimaliaDipteraLimoniidae

91E53C16-1E65-5C36-B63D-8D7CA5D2D916

https://zoobank.org/A81EBFF3-65C9-4CC2-8881-5BE9CDEC0D60

Figs 8, 9

##### Type material.

***Holotype***: China – Yunnan Prov. • ♂; Gongshan, Dulongjiang; 22 May 2007; Xingyue Liu (light trap); CAU.

##### Diagnosis.

Yellowish species. Prescutum and presutural scutum polished, with one brownish-yellow median stripe and two pairs of lateral brownish spots; two large brownish spots on scutal lobe. Legs mostly yellow, slightly darker on tips of tibiae and tarsi, femora without subterminal ring. Wing unpatterned. Abdomen mainly brownish yellow. Outer gonostylus blade-shaped, narrowing towards apex. Inner gonostylus dark, distinctly bifid; posterior branch longer than anterior branch, long curved spine-like.

##### Description.

**Male**. Body length 5.5 mm, wing length 6.2–6.5 mm. Antenna length 1.6 mm.

**Figure 8.**
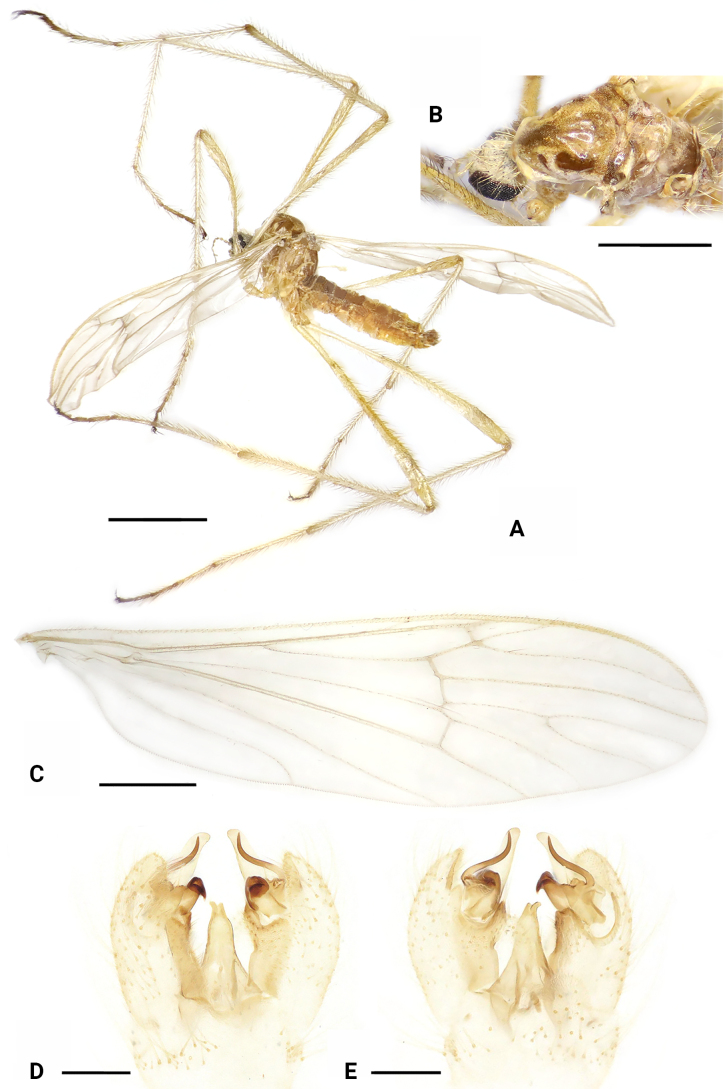
*Dasymallomyia immaculata* sp. nov. **A**. Male habitus, lateral view; **B**. Head and thorax, dorsal view; **C**. Right wing; **D**. Hypopygium, dorsal view; **E**. Hypopygium, ventral view. Scale bars: 2.0 mm (**A**); 1.0 mm (**B, C**); 0.2 mm (**D, E**).

***Head*** (Fig. 8A, B). Mostly yellowish with pale gray microtrichia. Setae on head brownish yellow to yellow. Antenna brownish yellow except distal flagellomeres yellow. Scape cylindrical, nearly 1.5 times as long as wide; flagellum 14-segmented with long verticils, about 2–3 times as long as segment; flagellomeres oval. Proboscis brownish yellow with brownish setae; palpi brownish yellow with brownish setae.

***Thorax*** (Fig. 8A, B). General yellow, distinctly polished on prescutum and scutum. Pronotum yellow. Prescutum and presutural scutum yellow with a dark median stripe and two pairs of brownish spots near median stripe; middle stripe distinct on anterior part, posterior part vague, nearly reaching transverse suture; anterior lateral spots distinctly smaller than posterior ones; posterior lateral spots subtriangular, separated with anterior lateral spots. Scutum yellow, with two large subtriangular brownish yellow spots on each scutal lobe, separated from or partly fused with each other, posterior one distinctly smaller than anterior one. Scutellum brownish yellow. Mediotergite brownish yellow, with small yellow area at lateral margin. Pleura brownish to brownish yellow on propleuron, anepisternum, ventral half of katepisternum, meron, and ventral part of laterotergite. Setae on thorax yellow. Legs mostly yellow; trochanters mainly brownish yellow; tips of tibiae and tarsi brownish. Setae on legs brownish yellow to yellow. Wing (Fig. 7C) hyaline, slightly tinged with yellowish, unpatterned. Veins mainly yellow, yellowish on Sc and veins near wing base. Venation: R_2_ ending at fork of R_3+4_; R_3_ about 1/3 as long as R_4_; cell m_1+2_ about as long as its petiole. Halter 0.8 mm long, pale yellow.

***Abdomen*** (Fig. 8A). Brownish on tergites and yellow on sternites, posterior margins of tergites weakly yellowish.

**Figure 9.**
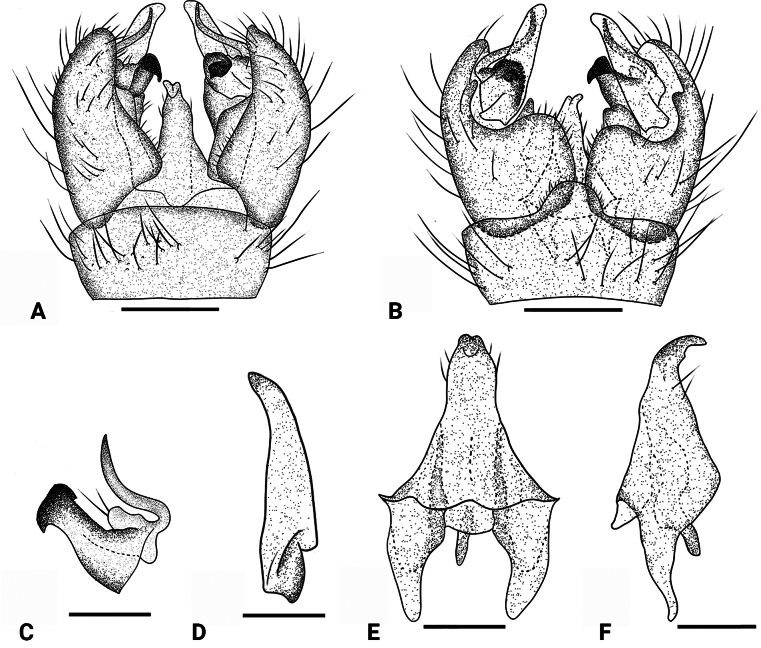
*Dasymallomyia immaculata* sp. nov. **A**. Hypopygium, dorsal view; **B**. Hypopygium, ventral view; **C**. Inner gonostylus; **D**. Outer gonostylus; **E**. Aedeagal complex, dorsal view; **F**. Aedeagal complex, lateral view. Scale bars: 0.2 mm (**A, B**); 0.1 mm (**C**–**F**).

***Hypopygium*** (Figs 8D, 8E, 9). Posterior margin of tergite 9 nearly straight (Fig. 9A). Sternite 9 strongly convex at middle (Fig. 9B). Gonocoxite longer than tergite 9, broad on basal part, rounded at tip (Figs 8D, 8E, 9A, 9B). Outer gonostylus blade-shaped, wide at base, and narrowing towards apex (Fig. 9D). Inner gonostylus distinctly bifid (Fig. 9C); anterior branch apically blackened, sclerotized, curved outward; posterior branch long curved spine-like; a rounded lobe near posterior branch. Aedeagus (Fig. 9E, F) subconical, its short, darkened tip directed posteroventrally, with some setae near middle.

**Female**. Unknown

##### Distribution.

China (Yunnan).

##### Etymology.

The specific name refers to the unpatterned wing.

##### Remark.

This new species is distinctive in having the femoral subterminal ring inconspicuous or absent. It is similar to *D. tanyphallus* Alexander, 1964 from India (Sikkim) in having a similar outer gonostylus, but can be distinguished from the latter by the aedeagus having a very short terminal point. In contrast, the aedeagus of *D. tanyphallus* bears an elongate terminal point (Alexander 1964: fig. 18).

## Supplementary Material

XML Treatment for Dasymallomyia bifurcata

XML Treatment for Dasymallomyia curvispina

XML Treatment for Dasymallomyia dentata

XML Treatment for Dasymallomyia immaculata

## Additional information

### Use of AI

No use of AI was reported.

### Author ORCIDs

Leyou Zhang https://orcid.org/0009-0001-5405-7710

Dawei Hong https://orcid.org/0000-0003-4645-3809

Bing Zhang https://orcid.org/0000-0002-3996-1760

Ding Yang https://orcid.org/0000-0002-7685-3478

### Data availability

All of the data that support the findings of this study are available in the main text.
